# Prevalence and effect of pre-treatment drug resistance on the virological response to antiretroviral treatment initiated in HIV-infected children – a EuroCoord-CHAIN-EPPICC joint project

**DOI:** 10.1186/s12879-016-1968-2

**Published:** 2016-11-08

**Authors:** Nicole Ngo-Giang-Huong, Linda Wittkop, Ali Judd, Peter Reiss, Tessa Goetghebuer, Dan Duiculescu, Antoni Noguera-Julian, Magdalena Marczynska, Carlo Giacquinto, Luminita Ene, Jose T. Ramos, Cristina Cellerai, Thomas Klimkait, Benedicte Brichard, Niels Valerius, Caroline Sabin, Ramon Teira, Niels Obel, Christoph Stephan, Stéphane de Wit, Claire Thorne, Diana Gibb, Christine Schwimmer, Maria Athena Campbell, Deenan Pillay, Marc Lallemant, The EuroCoord-CHAIN-EPPICC joint project study group

**Affiliations:** 1 IRD UMI 174 - PHPT-Faculty of Associated Medical Sciences, Chiang Mai University, 110, Intrawarorot Road, Sripoom, Muang, Chiang Mai 50200 Thailand; 2 Harvard T.H. Chan School of Public Health, Boston, USA; 3 Univ. Bordeaux, ISPED; INSERM, Centre INSERM U1219; CHU de Bordeaux, Pole de Sante Publique, F-33000 Bordeaux, France; 4 Medical Research Council Clinical Trials Unit, University College London, London, UK; 5 Academic Medical Centre, University of Amsterdam, Amsterdam, Netherlands; 6 Hôpital Saint-Pierre, Brussel, Belgium; 7 “Dr. Victor Babes” Hospital for Infectious and Tropical Diseases, Bucharest, Romania; 8 Sant Joan de Déu Hospital, University of Barcelona, Barcelona, Spain; 9 Medical University of Warsaw, Warsaw, Poland; 10 University of Padua, Padua, Italy; 11 “Dr. Victor Babes” Hospital for Infectious and Tropical Diseases, Bucharest, Romania; 12 University Hospital of Getafe, Madrid, Spain; 13 Lausanne University Hospital, Lausanne, Switzerland; 14 University of Basel, Basel, Switzerland; 15 Saint-Luc, Brussels, Belgium; 16 Hvidovre Hospital, University of Copenhagen, Copenhagen, Denmark; 17 University College London, London, UK; 18 Hospital de Sierrallana, Torrelavega, Spain; 19 Rigshospitalet, Copenhagen University Hospital, Copenhagen, Denmark; 20 University Clinic Frankfurt, Frankfurt, Germany; 21 AIDS Reference Center, CHU Saint-Pierre, Brussels, Belgium; 22 University College London, Institute of Child Health, London, UK; 23 Medical Research Council Clinical Trials Unit, London, UK; 24 Bordeaux RCC, INSERM, U897, Bordeaux, France; 25 Copenhagen RCC, Rigshospitalet, Copenhagen, Denmark

**Keywords:** HIV, Children, Pre-treatment drug resistance mutations, Virological failure, First-line combination antiretroviral therapy

## Abstract

**Background:**

Few studies have evaluated the impact of pre-treatment drug resistance (PDR) on response to combination antiretroviral treatment (cART) in children. The objective of this joint EuroCoord-CHAIN-EPPICC/PENTA project was to assess the prevalence of PDR mutations and their association with virological outcome in the first year of cART in children.

**Methods:**

HIV-infected children <18 years initiating cART between 1998 and 2008 were included if having at least one genotypic resistance test prior to cART initiation. We used the World Health Organization 2009 resistance mutation list and Stanford algorithm to infer resistance to prescribed drugs. Time to virological failure (VF) was defined as the first of two consecutive HIV-RNA > 500 copies/mL after 6 months cART and was assessed by Cox proportional hazards models. All models were adjusted for baseline demographic, clinical, immunology and virology characteristics and calendar period of cART start and initial cART regimen.

**Results:**

Of 476 children, 88 % were vertically infected. At cART initiation, median (interquartile range) age was 6.6 years (2.1–10.1), CD4 cell count 297 cells/mm^3^ (98–639), and HIV-RNA 5.2 log_10_copies/mL (4.7–5.7). Of 37 children (7.8 %, 95 % confidence interval (CI), 5.5–10.6) harboring a virus with ≥1 PDR mutations, 30 children had a virus resistant to ≥1 of the prescribed drugs. Overall, the cumulative Kaplan-Meier estimate for virological failure was 19.8 % (95 %CI, 16.4–23.9). Cumulative risk for VF tended to be higher among children harboring a virus with PDR and resistant to ≥1 drug prescribed than among those receiving fully active cART: 32.1 % (17.2–54.8) versus 19.4 % (15.9–23.6) (*P* = 0.095). In multivariable analysis, age was associated with a higher risk of VF with a 12 % reduced risk per additional year (HR 0.88; 95 %CI, 0.82–0.95; *P* < 0.001).

**Conclusions:**

PDR was not significantly associated with a higher risk of VF in children in the first year of cART. The risk of VF decreased by 12 % per additional year at treatment initiation which may be due to fading of PDR mutations over time. Lack of appropriate formulations, in particular for the younger age group, may be an important determinant of virological failure.

**Electronic supplementary material:**

The online version of this article (doi:10.1186/s12879-016-1968-2) contains supplementary material, which is available to authorized users.

## Background

Pre-treatment drug resistance (PDR) mutations have been demonstrated to be a major reason for virological failure (VF) after starting combination antiretroviral treatment (cART) in HIV-infected adults [1–6]. It is likely that they play a similar role in HIV-infected children. In vertically infected children, drug resistance mutations can be present prior to antiretroviral treatment due to the transmission of the resistant virus (transmitted drug resistance) from mothers [7] or the emergence of resistance mutations in wild-type virus transmitted from mothers as a consequence of pressure of maternal antiretroviral prophylaxis (drugs that have crossed the placenta and have a long half-life in infants) or neonatal prophylaxis [8–10].

In vertically infected children, exposure to single-dose nevirapine given for prevention of mother to child transmission (PMTCT) is associated with reduced efficacy of this drug when used for early treatment [11], and the response has been correlated with pre-existing resistance mutations [12–14]. Current treatment guidelines in the United States and Europe recommend genotypic resistance testing in all antiretroviral naive patients, including vertically infected children, to detect the presence of PDR mutations and to adapt their first-line treatment accordingly [15, 16]. Several studies have evaluated the prevalence of PDR mutations among HIV-infected children [6, 17–25]; however, few have determined their effect on virological response to first-line cART [6, 14, 23].

We assessed the prevalence of PDR mutations and their association with virological outcome in the first year of cART in children within a large collaboration of HIV observational cohort studies (EuroCoord-CHAIN-PENTA-EPPICC) in Europe and Thailand.

## Methods

### Study population

This study was conducted under the joint efforts of the Collaborative HIV and Anti-HIV Drug Resistance Network (CHAIN) and the EuroCoord network (CASCADE, COHERE, EuroSIDA and PENTA/EPPICC).

Cohorts participating through the EuroCoord network submitted defined dataset (patient demographics, use of cART, CD4 counts and HIV RNA measurements up to 16 months post-cART start, clinical (AIDS and death) events and genotypic resistance test) to their network-specific Coordination Centre, using the HIV Cohort Data Exchange Protocol [26].

Ethics approval was granted by the ethic committees of each of the participating cohorts according to local regulations. Written informed consent from participants’parents or legal guardians was obtained by the participating cohorts according to national legal and ethics requirements.

Maternal and infant prophylaxis data were not available for this analysis. Over the period of the study, no specific PMTCT protocol was recommended in Europe however some countries had their own PMTCT guidelines [27–31]. Practically, most cohorts used NVP or PI-based highly active antiretroviral therapy as the standard of care for mothers and 4–6 weeks zidovudine prophylaxis for infants. Zidovudine (ZDV) monotherapy with pre-labour caesarean section was an alternative for pregnant women who did not require treatment for their disease or in case of low viral load HIV. Threshold of CD4 varied over time and across countries (200 or 350 cells/mm^3^) as well as time to start antiretroviral prophylaxis. In Thailand national antiretroviral prophylaxis regimens have evolved from ZDV monotherapy for mothers and infants in 2000 to ZDV monotherapy plus single dose nevirapine (SD-NVP) in 2004 then to NVP-based cART for women with CD4 < 200 cells/mm^3^ or ZDV/SD-NVP for women with CD4 > 200 cells/mm^3^ with a 7-day post-partum tail regimen of ZDV/lamivudine to prevent NVP resistance in SD-NVP exposed women [32].

HIV-infected children aged <18 years were included in this study if they started cART between January 1,1998, and December 31, 2008 and if they had ≥1 sample for a genotypic test taken before the initiation of cART, whereby cART was defined as receiving at least three antiretroviral drugs. Genotypic resistance tests could have been performed retrospectively at each virology lab, i.e. test results may not necessarily have been used to guide first-line treatment. HIV genotyping assays were based on population sequencing techniques. We obtained nucleotide sequences for 93 % and amino-acid sequences for 7 % of the children. Alignment of nucleotide sequences was done centrally at the Copenhagen regional coordinating centre. If more than one genotypic test result was available mutations were cumulated.

### Statistical analyses

#### Definition of PDR mutations and resistance

PDR mutations and resistance were defined in two steps. First, the World Health Organization 2009 list of NRTI, NNRTI and PI mutations for the surveillance of transmitted drug resistant HIV strains [33] was used to identify PDR mutations and distinguish children harbouring a virus with ≥1 PDR mutations and those harbouring a virus with no PDR mutation, referred to as ‘no PDR’.

Second, for children harbouring a virus with ≥1 PDR mutations, the Stanford algorithm version 6.0.5 [34] was used to classify children into 2 groups: children receiving fully active cART (Stanford levels 1 and 2, corresponding to susceptible and potential low-level resistance, for all prescribed drugs) and those harbouring resistant HIV strain (Stanford levels 3 4, 5 corresponding to low-level, intermediate and high-level resistance, respectively) affecting ≥1 of the prescribed drugs. Among children with PDR mutations, those receiving fully active cART were referred to as ‘PDR and fully-active cART’ and children with a resistant strain were referred to ‘PDR and resistant’. Children with PDR receiving a fully active cART were regrouped with children harbouring a virus without PDR for the analysis of virological failure because there was no event among those receiving a fully active cART. Thus, two groups were considered: one group including all children with no PDR or ‘PDR and fully-active cART’ and one group including all children ‘PDR and resistant’.

#### Virological response

Virological failure was defined as the first of two consecutive viral loads >500 copies/mL after 6 months of cART (window 6–16 months), considering the date of first viral load >500 copies/mL as failure date. Children were censored if they died, stopped cART or were lost-to-follow-up. The time to virological failure was described by Kaplan-Meier curves and analysed by Cox proportional Hazards models. Baseline is defined as date of cART initiation.

All multivariable models were adjusted for the following potential confounders chosen a priori: sex, age, pre-treatment viral load (log_10_ transformed) and CD4 count, subtype (B, non B, unknown), region of origin (African, European, Asian, other/unknown), year of treatment start (1998–1999, 2000–2002, 2003–2004, 2005–2006, 2007–2008), previous AIDS diagnosis (yes, no, unknown) and HIV transmission risk group (vertical, heterosexual, injection drug use, other/unknown) and initial cART regimen (NNRTI plus ≥ 2NRTIs, boosted PI plus ≥ 2NRTIs, unboosted PI plus ≥ 2NRTIs, other). We conducted a stratified analysis according to initial cART regimen (NNRTI plus ≥ 2NRTIs, unboosted PI plus ≥ 2NRTIs). Proportionality assumptions were checked graphically by depicting the (log-log (Survival Probability) according to log(survival time) and by testing an interaction term between the covariables and the survival time. Analyses were performed using SAS 9.2 and 9.3 (SAS Institute, Inc., Cary, NC). *P* < 0.05 was considered significant.

#### Posteriori power calculation

Based on the number of virological failures observed among the 476 HIV-infected children, we could achieve 90 % power in a two-sided test with type I error of 5 % to detect a hazard ratio (HR) of 1.95 if risk groups were well balanced (i.e. 50 % of patients with high risk and 50 % with low risk). The HR limit would be 2.17, 3.05 and 4.64 if risk groups were not balanced whatever the direction (25–75, 10–90 and 5–95 % respectively) [35].

## Results

### Study population and baseline characteristics

A total of 476 children had sufficient follow-up and resistance data to be included in the analysis. They were enrolled in 18 cohort studies in 11 countries. At baseline, 246 children (51.7 %) were female and a large majority was infected through vertical transmission of HIV (Table 1). The median age at cART initiation was 6.6 years (interquartile range (IQR), 2.1–10.1), median baseline CD4 cell count 297 cells/mm^3^ (IQR, 98–639), and median HIV RNA load 5.2 log_10_ copies/mL (IQR, 4.7–5.7) (Table 1).

**Table 1:** Characteristics at cART initiation

Characteristics		Number	Percent
Sex	Female	246	51.7
Age years median (IQR)		6	(2 ; 10)
Age (years)	<2	115	24.2
	2–5	113	23.7
	6–12	197	41.4
	13–17	51	10.7
Region of origin	Africa	113	23.7
	Asia	194	40.8
	Europe	118	24.8
	Other/unknown	51	10.7
Transmission risk group	Vertical	419	88.0
	IDU	1	0.2
	Heterosexual	12	2.5
	Other/unknown	44	9.2
Previous AIDS diagnosis	Yes	99	20.8
	No	375	78.8
	Unknown	2	0.4
Pretreatment CD4 cell count (/mm3) median (IQR)*		364	(294; 422)
Pretreatment CD4 cell count (/mm^3^)*	<200	156	37.9
	≥200 and < 350	77	19.2
	≥350 and <500	41	10.0
	≥500	138	33.5
Pretreatment HIV RNA (log_10_ copies/mL) median (IQR)**		5.2	(4.7; 5.7)
Pretreatment HIV RNA (log_10_ copies/mL)**	<4	36	8
	≥4 and <5	137	30.5
	≥5 and <6	214	47.7
	>6	62	13.8
HIV subtype	Non B	382	80.3
	B	63	13.2
	Unknown	31	6.5
Year of cART start	1998–1999	105	22.1
	2000–2002	57	12.0
	2003–2004	112	23.5
	2005–2006	149	31.3
	2007–2008	53	11.1
Antiretroviral drug combination	NNRTI plus ≥ 2NRTIs^a^	273	57.4
	Unboosted PI plus ≥ 2NRTIs^b^	139	29.2
	Boosted PI plus ≥ 2NRTIs^c^	43	9.0
	Other^d^	21	4.4

^a^Includes 232 who received 2 NRTIs and 41 who received 3 NRTIs. Overall, 184 were on efavirenz and 89 on nevirapine

^b^Includes 136 who received 2 NRTIs and 1 each who received 3, 4, and 5 NRTIs. 131 were on nelfinavir, 3 on indinavir, and 2 on ritonavir

^c^Includes 37 who received 2 NRTIs, 5 who received 3 NRTIs, and 1 who received 4 NRTIs. 32 were on lopinavir/ritonavir, 5 on fosamprenavir/ritonavir, 4 on indinavir/ritonavir, and one each on atazanavir or saquinavir with ritonavir

^d^Includes 10 who received (1 NNRTI plus ≥ 2NRTIs plus 1 PI or 1 boosted PI), 5 who received (2NNRTIs plus ≥ 2NRTIs), 4 who received (≥1 NRTI plus 2 PI/boosted PI), 1 who received (1 NRTI plus boosted PI plus integrase inhibitor plus fusion inhibitor), and 1 who received (1 NRTI plus 1 boosted PI plus 1 fusion inhibitor). *CD4 cell count measurements at cART initiation were available for 412 children. **Viral load measurements at cART initiation were available for 449 children

Two hundred thirty two (48.7 %) children initiated cART with 2 nucleoside reverse transcriptase inhibitors (NRTIs) and 1 nonnucleoside reverse transcriptase inhibitor (NNRTI) and 41 (8.6 %) with 3 NRTIs and 1 NNRTI, 139 (29.2 %) with 2 or more NRTIs and 1 unboosted protease inhibitor (PI) (mostly nelfinavir), 43 (9 %) with 2 or more NRTIs and 1 boosted PI, and 21 (4.8 %) with other combinations of three or more drugs (Table 1).

### Pre-treatment resistance mutations

At least one PDR mutation was identified in 37 children (7.8 %; 95%CI, 5.5–10.6) of whom 76 % harbored a virus with NRTI, 46 % with NNRTI and 8 % with PI resistance mutations and 30 % had PDR mutations to 2 ARV classes (Table 2). The proportion of children with PDR mutations was higher among children below 2 years of age than among those aged 2 years or more; 12.2 % (14 of 155) versus vs 6.4 % (23 of 361) (*P* = 0.043).

**Table 2:** Prevalence of resistance mutations using the WHO 2009 list for surveillance of transmitted drug resistance mutations

	Number	Percent	95 % CI
At least 1 resistance mutation	37	7.8	5.5–10.6
≥ 1 NRTI resistance mutation	28	5.9	3.9–8.4
≥ 1 NNRTI resistance mutation	17	3.6	2.1–5.7
≥ 1 PI resistance mutation	3	0.6	0.1–1.8
Resistance mutations to 2 ARV classes	11	2.3	1.2–4.1
Resistance mutations to 3 ARV classes	0	0	

Of these 37 children, seven were in the ‘PDR and fully-active cART’ group and 30 children in the ‘PDR and resistant’ group. In this latter group, 11 children had PDR mutations to 2 antiretroviral drug classes (Table 2). Prevalence of PDR mutations was 7.1, 7.7, 7.8 and 9.8 % in children from African, European, Asian or unknown origin. There was no statistical difference of PDR according the geographic origin (*P* = 0.93). Furthermore, baseline characteristics were not different between children with and without PDR mutations (Additional file 1: Table S1).

### Analysis of virological failure and risk factors

**Fig. 1:**
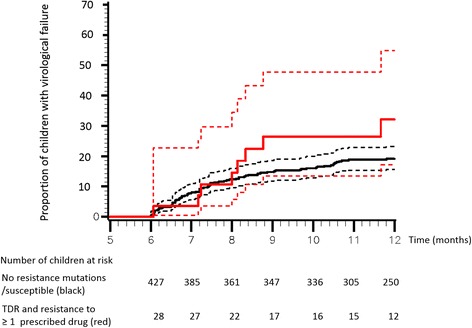
Kaplan-Meier estimate for proportion of children with virological failure. Risk of failure in children harboring virus fully “susceptible” to cART (black continuous line) versus risk of failure in children with virus “resistant” to at least one drug (red continuous line) (Log-rank test: *P* = 0.0954). The dotted lines correspond to 95 % confidence intervals. In total, 21 patients stopped cART between cART initiation and 6 months of follow-up and were censored at cART stop. This explains why at 6 months there are 427 participants at risk in the no PDR/PDR and susceptible group and 28 participants at risk in the PDR and resistant group

Overall, median follow-up was 12 months (10; 14) corresponding to 449 person years (PY) of follow-up; children with fully active treatment were followed-up over a median of 12 (10; 14) months (424 PY of follow-up), and children with PDR and receiving a treatment to which the virus was resistant were followed over a median of 11 (8; 13) months (26 PY of follow-up). Median number of viral load measurements after baseline was 4 (IQR: 2; 8) and there was no difference in median viral load measurements between the group no PDR/PDR susceptible and the group PDR and resistant with 4 (2;8) and 4 (2;7) measurements, respectively. Virological failure occurred in 83 of 446 children who started with fully active cART (no PDR or having “PDR and fully-active cART”) and in 9 of 30 children of the “PDR and resistant” group. The overall estimated cumulative Kaplan-Meier risk of virological failure at 12 months of cART was 19.8 % (95%CI, 16.4–23.9). Children in the ‘PDR and resistant’ group tended to have higher cumulative risk of virological failure than those with no PDR or having ‘PDR and fully-active cART’, at 32.1 % (17.2–54.8) versus 19.4 % (15.9–23.6) (*p* = 0.095) (Fig. 1). Crude incidence rates of virological failure were 35 per 100 PY of follow-up in the ‘PDR and resistant’ group compared to 19 per 100 PY of follow-up in the no PDR/‘PDR and fully active cART’ group.

In univariable analysis, younger age, year at cART initiation (1998–1999 vs 2007–2008), initial cART regimen (Unboosted PI plus ≥ 2NRTIs vs NNRTI plus ≥ 2NRTIs) and region of origin (African vs Europe) were significantly associated with VF (Table 3). PDR and resistance to ≥ 1 prescribed antiretroviral drugs was not significantly associated with a higher risk of virological failure in univariable analysis (HR, 1.78; 95 % CI, 0.89–3.54; *P* = 0.101) and also after adjustment for covariables (aHR, 1.42; 95 % CI, 0.62–3.24; *P* = 0.400).

**Table 3:** Univariable and multivariable analysis of risk factors for virological failure

	Univariable Analysis				Multivariable Analysis			
	HR	(95 % CI)	*P*	Global P	aHR	(95 % CI)	P	Global P
PDR resistant vs no PDR/susceptible	1.78	(0.89–3.54)	0.101		1.42	(0.62–3.24)	0.400	
Sex (male vs female)	1.06	(0.71–1.60)	0.770		1.45	(0.91–2.31)	0.117	
Age (per additional year)	0.89	(0.84–0.93)	0.000		0.88	(0.82–0.95)	0.0005	
Origin (ref Europe)				0.029				0.720
Africa	0.40	(0.20–0.78)	0.008		1.14	(0.49–2.62)	0.759	
Asia	0.73	(0.45–1.18)	0.197		1.02	(0.464–2.25)	0.960	
Other/unknown	1.10	(0.58–2.09)	0.764		0.65	(0.28–1.50)	0.313	
Previous AIDS diagnosis (yes vs no)	1.18	(0.73–1.91)	0.495		1.00	(0.56–1.76)	0.986	
Pretreatment CD4 count (per 100 cells/mm^3^)	1.01	(0.98–1.04)	0.510		0.96	(0.92–1.00)	0.048	
Pretreatment viral load (per 1 log_10_ copies/mL increase)	1.16	(0.90–1.50)	0.254		0.99	(0.76–1.28)	0.987	
Subtype (non B vs B)	0.85	(0.48–1.50)	0.569		0.70	(0.34–1.45)	0.335	
Year of treatment start (ref: 2007–2008)				0.018				0.818
1998–1999	3.14	(1.22–8.07)	0.018		1.11	(0.25–4.95)	0.891	
2000–2002	2.01	(0.71–5.70)	0.190		1.89	(0.48–7.40)	0.361	
2003–2004	1.97	(0.75–5.21)	0.170		1.29	(0.34–4.96)	0.710	
2005–2006	1.36	(0.52–3.60)	0.532		1.27	(0.34–4.79)	0.7230	
Antiretroviral drug combination (ref: NNRTI plus ≥ 2NRTIs)				0.0002				0.007
Unboosted PI plus ≥ 2NRTIs	19.5	(2.68; 1.73)	<0.001		3.65	(1.65; 8.05)	0.001	
Boosted PI plus ≥ 2NRTIs	1.26	(0.56; 2.83)	0.572		1.79	(0.76; 4.57)	0.222	
Other	1.73	(0.62; 4.85)	0.300		2.65	(0.90; 7.81)	0.077	

All multivariable models were adjusted for sex, age, pre-treatment viral load (log_10_ transformed) and CD4 count, subtype (B, non B, unknown), region of origin (African, European, Asian, other/unknown), year of treatment start (1998–1999, 2000–2002, 2003–2004, 2005–2006), previous AIDS diagnosis (yes, no, unknown) and HIV transmission risk group (heterosexual, injection drug use, perinatal, other/unknown), initial antiretroviral drug combination

*HR* hazard ratio

Upon multivariable analysis, age remained associated with VF and the risk of VF decreased by 12 % per year older at cART initiation (HR 0.88; 95 %CI, 0.82–0.95; *P* < 0.001). The association between initial cART regimen and virological failure persisted in the multivariable model. In stratified analysis according to the initial treatment regimen, the association between PDR and resistance to ≥ 1 prescribed antiretroviral drugs was not significantly associated with a higher risk of virological failure neither in the > =2NRTI + 1NNRTI stratum nor in > =2NRTIs + an unboosted PIs stratum (Additional file 2: Figure S1).

## Discussion

In this large international pediatric multicohort analysis, the prevalence of PDR mutations in antiretroviral naïve children was 7.8 % (95 %CI, 5.5–10.6) and was similar to that reported in a large European multicohort study, 9.5 % (95 % CI, 8.9–10.1) of 10,056 patients, mostly adults [6]. Most of mutations were associated with resistance to NRTIs and NNRTIs reflecting the drug classes used over that period. Other studies conducted in different settings with smaller population size reported a frequency of drug resistance mutations ranging between 5.7 and 100 % depending on the age of children at time of testing, the birth period and the genotyping technique sensitivity [7, 9, 10, 12, 17–25].

Cumulative incidence of virological failure tended to be higher among children starting cART with PDR and resistance ≥1 drug prescribed than among those starting with a fully active regimen (Log-rank test: *P* = 0.095) but no direct association between PDR mutations and VF was found, in contrast to what was reported in the large European multicohort study [6]. This may be a consequence of the smaller sample size of our population and the relatively low prevalence of PDR mutations. Based on our population, we could achieve 75 % power to detect a HR of 3.13 [35]. Another possible reason may have been the long time interval (6 years or more in over half the population) between perinatal infection and the onset of treatment. In this study, higher frequency of PDR mutations was indeed observed in children less than 2 years. During this interval, minor populations of drug-resistant virus, in particular populations that might emerge following maternal or neonatal prophylaxis with either zidovudine as monotherapy or single-dose nevirapine, might have diminished or disappeared altogether, so that individuals with minor resistant variants might have been categorized as entirely without mutations [12, 36, 37]. Indeed high-sensitivity methods for measurement of drug resistance were not used in this study. However the usefulness of using high-sensitivity methods over consensus sequencing to predict virological failure in children is unclear [12, 13].

High virological failure rates on first-line cART have been reported in children in different settings [17, 38–42], including within clinical trials [11, 43, 44]. Among independent predictors of virological failure identified were use of nevirapine vs efavirenz or ritonavir [41, 43–46], poor adherence to ART [47], prior exposure to single dose of nevirapine presence of baseline resistance [12, 14], and younger age [39]. In our study VF was more likely to occur when cART was started at younger ages (HR 0.88). This association of outcome with age is probably due to several factors. Treatment of younger children during the decade that ARVs were started depended heavily on liquid forms of ARVs that, particularly for the more potent protease inhibitors, are often unpalatable. Also during the time of this study information about the pharmacokinetics of several important drugs, particularly nevirapine and nelfinavir, was limited in children aged less than 2 years and required dose adjustment [48, 49]. Fixed drug combinations suitable for children were not available during the period of this study, so large pill burdens or unpalatable liquid formulations [50] were a common problem in pediatrics [51–54]. For those reasons, adherence and therefore efficacy of cART were often low. The risk of virological failure is correlated with the proportion of missed doses, but impact of nonadherence on viral resistance depends on pharmacology (regimen potency, pharmacokinetics, drug interactions) and viral (viral fitness, and resistance barrier to ARV) factors. Suboptimal or poor adherence (missed/late doses) to drugs can result in sub-therapeutic plasma concentrations of drugs and subsequent development of drug resistance to one or more drugs in a given regimen, and possibly cross-resistance to other drugs of the same class. In the absence of drug pressure as in treatment interruptions or discontinuations, resistance mutations are unlikely to be selected.

Initial cART regimen was significantly associated with virological failure, however these results should be interpreted with caution as children were not randomized to initial treatment and thus results may be subject of indication bias, e.g. children with more advanced disease status (higher proportion of children with a previous AIDS diagnosis) were more likely to receive boosted PI regimen.

Our findings of higher risk of VF in younger children support the 2013 WHO recommendation to treat all children <3 years of age with boosted-PI [27]. In addition, due to the higher barrier to drug resistance of boosted PIs the effect of PDR might be of limited importance today for clinical practice. Nevertheless pre-existing NNRTI resistance may still compromise response to PI-based first line cART. Indeed PI resistance can emerge in children with PDR as evidenced by the presence of multidrug resistance mutations in single HIV genome in children failing PI-based first line [55].

Our study has some limitations. It was an observational study and some resistance results were obtained retrospectively. However, all children starting cART with a sample for HIV genotypic resistance testing were included in the analysis and testing was performed with no knowledge of the outcome. Association of outcome with age may be different at the era of WHO PMTCT option B+. Indeed, in that context more pregnant women receive NNRTI-based cART and children who may become infected with resistant virus selected during treatment failure may have NNRTI resistance mutations that can persist longer than those selected by single dose-nevirapine. Another limitation is that we were not able to assess the effect of the pretreatment CD4 percentage since this was measured in only 49 % of children and to address adherence and specific HIV subtypes effect. Lastly, despite the large study population it was not possible to specifically analyze more substrata as the power was already limited for the overall question of this analysis.

## Conclusions

Our study shows that the risk of VF decreased with age at treatment initiation. We hypothesize that this finding might be due to fading of PDR mutations over time although the direct association between those resistance mutations and VF did not reach statistical significance potentially due to lack of power. Furthermore, misclassification due to the relatively low sensitivity of population sequencing in detecting mutations in viral subpopulations long after initial infection or exposure to ARVs for the prevention of mother to child transmission shortly prior to infection could have occurred diluting the effect. Also, lack of appropriate formulations, in particular for the younger age group, which may possibly have resulted in poorer adherence, might be an important determinant of virological failure.

## Additional files

Additional file 1: Characteristics at cART initiation according to presence/absence of PDR. Table. (DOCX 20 kb)
Additional file 2: Crude Hazard Ratios (cHR) and adjusted Hazard Ratios (aHR) for the association between PDR and resistant group versus no PDR/susceptible group from Cox proportional Hazards models stratified by initial cART regimen. HR in the overall group are also presented in more details in Table 3 of the article. All models were adjusted for sex, age, pre-treatment viral load (log_10_ transformed) and CD4 count, subtype, region of origin, year of treatment start, previous AIDS diagnosis and HIV transmission risk group. (DOCX 46 kb)

## Abbreviations

cART: Combination antiretroviral treatment
HR: Hazard ratio
PDR: Pre-treatment drug resistance
PI: Protease inhibitor
PMTCT: Prevention of mother to child transmission
SD-NVP: Single dose nevirapine
VF: Virological failure
ZDV: Zidovudine

## References

[1] Geretti AM (2007). Epidemiology of antiretroviral drug resistance in drug-naive persons. Curr Opin Infect Dis.

[2] Goodman DD, Zhou Y, Margot NA, McColl DJ, Zhong L, Borroto-Esoda K, Miller MD, Svarovskaia ES (2011). Low level of the K103N HIV-1 above a threshold is associated with virological failure in treatment-naive individuals undergoing efavirenz-containing therapy. AIDS.

[3] Kantor R, Smeaton L, Vardhanabhuti S, Hudelson SE, Wallis CL, Tripathy S, Morgado MG, Saravanan S, Balakrishnan P, Reitsma M, et al. Pretreatment HIV Drug Resistance and HIV-1 Subtype C Are Independently Associated With Virologic Failure: Results From the Multinational PEARLS (ACTG A5175) Clinical Trial. Clin Infect Dis. 2015;60(10):1541-9. 10.1093/cid/civ102 PMC4425827 25681380

[4] Kuritzkes DR, Lalama CM, Ribaudo HJ, Marcial M, Meyer WA, Shikuma C, Johnson VA, Fiscus SA, D’Aquila RT, Schackman BR (2008). Preexisting resistance to nonnucleoside reverse-transcriptase inhibitors predicts virologic failure of an efavirenz-based regimen in treatment-naive HIV-1-infected subjects. J Infect Dis.

[5] Paredes R, Lalama CM, Ribaudo HJ, Schackman BR, Shikuma C, Giguel F, Meyer WA, Johnson VA, Fiscus SA, D’Aquila RT (2010). Pre-existing minority drug-resistant HIV-1 variants, adherence, and risk of antiretroviral treatment failure. J Infect Dis.

[6] Wittkop L, Gunthard HF, de Wolf F, Dunn D, Cozzi-Lepri A, de Luca A, Kucherer C, Obel N, von Wyl V, Masquelier B (2011). Effect of transmitted drug resistance on virological and immunological response to initial combination antiretroviral therapy for HIV (EuroCoord-CHAIN joint project): a European multicohort study. Lancet Infect Dis.

[7] Zeh C, Weidle PJ, Nafisa L, Lwamba HM, Okonji J, Anyango E, Bondo P, Masaba R, Fowler MG, Nkengasong JN (2011). HIV-1 drug resistance emergence among breastfeeding infants born to HIV-infected mothers during a single-arm trial of triple-antiretroviral prophylaxis for prevention of mother-to-child transmission: a secondary analysis. PLoS Med.

[8] Eshleman SH, Mracna M, Guay LA, Deseyve M, Cunningham S, Mirochnick M, Musoke P, Fleming T, Glenn Fowler M, Mofenson LM (2001). Selection and fading of resistance mutations in women and infants receiving nevirapine to prevent HIV-1 vertical transmission (HIVNET 012). AIDS.

[9] Martinson NA, Morris L, Gray G, Moodley D, Pillay V, Cohen S, Dhlamini P, Puren A, Bhayroo S, Steyn J (2007). Selection and persistence of viral resistance in HIV-infected children after exposure to single-dose nevirapine. J Acquir Immune Defic Syndr.

[10] Masquelier B, Chaix ML, Burgard M, Lechenadec J, Doussin A, Simon F, Cottalorda J, Izopet J, Tamalet C, Douard D (2001). Zidovudine genotypic resistance in HIV-1-infected newborns in the French perinatal cohort. J Acquir Immune Defic Syndr.

[11] Lockman S, Shapiro RL, Smeaton LM, Wester C, Thior I, Stevens L, Chand F, Makhema J, Moffat C, Asmelash A (2007). Response to antiretroviral therapy after a single, peripartum dose of nevirapine. N Engl J Med.

[12] Hunt GM, Coovadia A, Abrams EJ, Sherman G, Meyers T, Morris L, Kuhn L (2011). HIV-1 drug resistance at antiretroviral treatment initiation in children previously exposed to single-dose nevirapine. AIDS.

[13] Lehman DA, Wamalwa DC, McCoy CO, Matsen FA, Langat A, Chohan BH, Benki-Nugent S, Custers-Allen R, Bushman FD, John-Stewart GC (2012). Low-frequency nevirapine resistance at multiple sites may predict treatment failure in infants on nevirapine-based treatment. J Acquir Immune Defic Syndr.

[14] MacLeod IJ, Rowley CF, Thior I, Wester C, Makhema J, Essex M, Lockman S (2010). Minor resistant variants in nevirapine-exposed infants may predict virologic failure on nevirapine-containing ART. J Clin Virol.

[15] Panel on Antiretroviral Therapy and Medical Management of HIV-Infected Children. Guidelines for the Use of Antiretroviral Agents in Pediatric HIV Infection. Available at https://aidsinfo.nih.gov/contentfiles/lvguidelines/pediatricguidelines.pdf. Accessed 16 Apr 2015. [Clinical and Laboratory Monitoring of Children with HIV Infection, Entry into Care—Baseline Evaluation: D-4]. Update March 5, 2015.

[16] Bamford A, Turkova A, Lyall H, Foster C, Klein N, Bastiaans D, Burger D, Bernadi S, Butler K, Chiappini E, et al. Paediatric European Network for Treatment of AIDS (PENTA) guidelines for treatment of paediatric HIV-1 infection 2015: optimizing health in preparation for adult life. HIV Med. 2015. doi:10.1111/hiv.12217. 10.1111/hiv.12217 PMC5724658 25649230

[17] Fokam J, Salpini R, Santoro MM, Cento V, Perno CF, Colizzi V, Ndumbe PM, Fokunang Ntungen C, Ndiang Tetang SM, Nanfack AJ (2011). Drug resistance among drug-naive and first-line antiretroviral treatment-failing children in Cameroon. Pediatr Infect Dis J.

[18] Neogi U, Sahoo PN, De Costa A, Shet A (2012). High viremia and low level of transmitted drug resistance in anti-retroviral therapy-naive perinatally-infected children and adolescents with HIV-1 subtype C infection. BMC Infect Dis.

[19] Gupta RK, Jordan MR, Sultan BJ, Hill A, Davis DH, Gregson J, Sawyer AW, Hamers RL, Ndembi N, Pillay D (2012). Global trends in antiretroviral resistance in treatment-naive individuals with HIV after rollout of antiretroviral treatment in resource-limited settings: a global collaborative study and meta-regression analysis. Lancet.

[20] Hamers RL, Wallis CL, Kityo C, Siwale M, Mandaliya K, Conradie F, Botes ME, Wellington M, Osibogun A, Sigaloff KC (2011). HIV-1 drug resistance in antiretroviral-naive individuals in sub-Saharan Africa after rollout of antiretroviral therapy: a multicentre observational study. Lancet Infect Dis.

[21] Parker MM, Wade N, Lloyd RM, Birkhead GS, Gallagher BK, Cheku B, Sullivan T, Taylor J (2003). Prevalence of genotypic drug resistance among a cohort of HIV-infected newborns. J Acquir Immune Defic Syndr.

[22] Perez L, Correa C, Campos YA, Gonzalez I, Perez J, Martinez PA, Alvarez A, Soto Y, Kouri V (2011). Drug-resistant HIV-1 in Cuban children and their seropositive mothers. MEDICC Rev.

[23] Rojas Sanchez P, Holguin A (2014). Drug resistance in the HIV-1-infected paediatric population worldwide: a systematic review. J Antimicrob Chemother.

[24] Simonetti SR, Schatzmayr HG, Simonetti JP (2003). Human immunodeficiency virus type 1: drug resistance in treated and untreated Brazilian children. Mem Inst Oswaldo Cruz.

[25] Soto-Ramirez LE, Rodriguez-Diaz R, Harris DR, Hazra R (2010). HIV Drug Resistance-Associated Mutations in Antiretroviral Naive HIV-1-Infected Latin American Children. Adv Virol.

[26] Kjaer J, Ledergerber B (2004). HIV cohort collaborations: proposal for harmonization of data exchange. Antivir Ther.

[27] Aebi-Popp K, Mulcahy F, Rudin C, Hoesli I, Gingelmaier A, Lyons F, Thorne C (2013). National Guidelines for the prevention of mother-to-child transmission of HIV across Europe - how do countries differ?. Eur J Public Health.

[28] British HIV Association. Guidelines for the management of HIV infection in pregnant women and the prevention of mother-to-child transmission of HIV BHIVA Pregnancy guidelines – March 2005. http://www.bhiva.org/documents/Guidelines/Pregnancy/2005/2005PregGL.pdf. Accessed 24 Mar 2016.

[29] Salzberger B, Marcus U, Vielhaber B, Arasteh K, Golz J, Brockmeyer NH, Rockstroh J (2004). German-Austrian recommendations for the antiretroviral therapy of HIV-infection (status May 2004). Eur J Med Res.

[30] Iribarren JA, Labarga P, Rubio R, Berenguer J, Miro JM, Antela A, Gonzalez J, Moreno S, Arrizabalaga J, Chamorro L (2004). Spanish GESIDA/Nacional AIDS Plan Recommendations for antiretroviral therapy in HIV-infected Adults (October 2004). Enferm Infecc Microbiol Clin.

[31] Iribarren JA, Ramos JT, Guerra L, Coll O, de Jose MI, Domingo P, Fortuny C, Miralles P, Parras F, Pena JM (2001). Prevention of vertical transmission and treatment of infection caused by the human immunodeficiency virus in the pregnant woman. Recommendations of the Study Group for AIDS, Infectious Diseases, and Clinical Microbiology, the Spanish Pediatric Association, the National AIDS Plan and the Spanish Gynecology and Obstetrics Society. Enferm Infecc Microbiol Clin.

[32] Voramongkol N, Naiwatanakul T, Punsuwan N, Kullerk N, Lolekha R, Sarika P, Pattarakulwanich S, McConnell MS (2013). Compliance with and outcomes of CD4-based national guidelines for prevention of mother-to-child transmission of HIV for Thailand, 2006–2007. Southeast Asian J Trop Med Public Health.

[33] Bennett DE, Camacho RJ, Otelea D, Kuritzkes DR, Fleury H, Kiuchi M, Heneine W, Kantor R, Jordan MR, Schapiro JM (2009). Drug resistance mutations for surveillance of transmitted HIV-1 drug-resistance: 2009 update. PLoS One.

[34] Liu TF, Shafer RW (2006). Web resources for HIV type 1 genotypic-resistance test interpretation. Clin Infect Dis.

[35] Schoenfeld DA (1983). Sample-size formula for the proportional-hazards regression model. Biometrics.

[36] Flys TS, McConnell MS, Matovu F, Church JD, Bagenda D, Khaki L, Bakaki P, Thigpen MC, Eure C, Fowler MG (2008). Nevirapine resistance in women and infants after first versus repeated use of single-dose nevirapine for prevention of HIV-1 vertical transmission. J Infect Dis.

[37] Fisher RG, Smith DM, Murrell B, Slabbert R, Kirby BM, Edson C, Cotton MF, Haubrich RH, Kosakovsky Pond SL, Van Zyl GU (2015). Next generation sequencing improves detection of drug resistance mutations in infants after PMTCT failure. J Clin Virol.

[38] Bunupuradah T, Puthanakit T, Kosalaraksa P, Kerr S, Boonrak P, Prasitsuebsai W, Lumbiganon P, Mengthaisong T, Phasomsap C, Pancharoen C (2011). Immunologic and virologic failure after first-line NNRTI-based antiretroviral therapy in Thai HIV-infected children. AIDS Res Ther.

[39] Bunupuradah T, Sricharoenchai S, Hansudewechakul R, Klinbuayaem V, Teeraananchai S, Wittawatmongkol O, Akarathum N, Prasithsirikul W, Ananworanich J (2015). Risk of first-line antiretroviral therapy failure in HIV-infected Thai children and adolescents. Pediatr Infect Dis J.

[40] Charpentier C, Gody JC, Mbitikon O, Moussa S, Matta M, Pere H, Fournier J, Longo Jde D, Belec L (2012). Virological response and resistance profiles after 18 to 30 months of first- or second-/third-line antiretroviral treatment: a cross-sectional evaluation in HIV type 1-infected children living in the Central African Republic. AIDS Res Hum Retroviruses.

[41] Jittamala P, Puthanakit T, Chaiinseeard S, Sirisanthana V (2009). Predictors of virologic failure and genotypic resistance mutation patterns in thai children receiving non-nucleoside reverse transcriptase inhibitor-based antiretroviral therapy. Pediatr Infect Dis J.

[42] Pham H, Ishizaki A, Nguyen L, Phan C, Phung T, Takemoto K, Pham A, Bi X, Khu D, Ichimura H. Two-year outcome of first-line antiretroviral therapy among HIV-1 vertically infected children in Hanoi, Vietnam. Int J STD AIDS. 2015;26(11):821-30. 10.1177/0956462414556328 25332224

[43] Palumbo P, Lindsey JC, Hughes MD, Cotton MF, Bobat R, Meyers T, Bwakura-Dangarembizi M, Chi BH, Musoke P, Kamthunzi P (2010). Antiretroviral treatment for children with peripartum nevirapine exposure. N Engl J Med.

[44] Violari A, Lindsey JC, Hughes MD, Mujuru HA, Barlow-Mosha L, Kamthunzi P, Chi BH, Cotton MF, Moultrie H, Khadse S (2012). Nevirapine versus ritonavir-boosted lopinavir for HIV-infected children. N Engl J Med.

[45] Lowenthal ED, Ellenberg JH, Machine E, Sagdeo A, Boiditswe S, Steenhoff AP, Rutstein R, Anabwani G, Gross R (2013). Association between efavirenz-based compared with nevirapine-based antiretroviral regimens and virological failure in HIV-infected children. JAMA.

[46] Penazzato M, Prendergast A, Tierney J, Cotton M, Gibb D (2012). Effectiveness of antiretroviral therapy in HIV-infected children under 2 years of age. Cochrane Database Syst Rev.

[47] Sebunya R, Musiime V, Kitaka SB, Ndeezi G (2013). Incidence and risk factors for first line anti retroviral treatment failure among Ugandan children attending an urban HIV clinic. AIDS Res Ther.

[48] Aboulker JP, Babiker A, Chaix ML, Compagnucci A, Darbyshire J, Debre M, Faye A, Giaquinto C, Gibb DM, Harper L (2004). Highly active antiretroviral therapy started in infants under 3 months of age: 72-week follow-up for CD4 cell count, viral load and drug resistance outcome. AIDS.

[49] Litalien C, Faye A, Compagnucci A, Giaquinto C, Harper L, Gibb DM, Jacqz-Aigrain E, Paediatric European Network for Treatment of AEC (2003). Pharmacokinetics of nelfinavir and its active metabolite, hydroxy-tert-butylamide, in infants perinatally infected with human immunodeficiency virus type 1. Pediatr Infect Dis J.

[50] World Health Organization. Consolidated guidelines on the use of antiretroviral drugs for treating and preventing HIV infection: recommendations for a public health approach June 2013. http://www.who.int/hiv/pub/guidelines/arv2013/en/. Accessed 27 Oct 2016.

[51] Davies MA, Boulle A, Fakir T, Nuttall J, Eley B (2008). Adherence to antiretroviral therapy in young children in Cape Town, South Africa, measured by medication return and caregiver self-report: a prospective cohort study. BMC Pediatr.

[52] Gibb DM, Goodall RL, Giacomet V, McGee L, Compagnucci A, Lyall H, Paediatric European Network for Treatment of ASC (2003). Adherence to prescribed antiretroviral therapy in human immunodeficiency virus-infected children in the PENTA 5 trial. Pediatr Infect Dis J.

[53] Reddington C, Cohen J, Baldillo A, Toye M, Smith D, Kneut C, Demaria A, Bertolli J, Hsu HW (2000). Adherence to medication regimens among children with human immunodeficiency virus infection. Pediatr Infect Dis J.

[54] Van Dyke RB, Lee S, Johnson GM, Wiznia A, Mohan K, Stanley K, Morse EV, Krogstad PA, Nachman S, ACTGASPACTGST Pediatric (2002). Reported adherence as a determinant of response to highly active antiretroviral therapy in children who have human immunodeficiency virus infection. Pediatrics.

[55] Lange CM, Hue S, Violari A, Cotton M, Gibb D, Babiker A, Otwombe K, Panchia R, Dobbels E, Jean-Philippe P (2015). Single Genome Analysis for the Detection of Linked Multiclass Drug Resistance Mutations in HIV-1-Infected Children After Failure of Protease Inhibitor-Based First-Line Therapy. J Acquir Immune Defic Syndr.

