# Supplementary material for: Prevalence and effect of pre-treatment drug resistance on the virological response to antiretroviral treatment initiated in HIV-infected children – a EuroCoord-CHAIN-EPPICC joint project
Source: BMC Infect Dis. 2016 Nov 8;16:654. doi: 10.1186/s12879-016-1968-2 (PMC5101717; doi:10.1186/s12879-016-1968-2)
Supplement: Additional file 1: — Characteristics at cART initiation according to presence/absence of PDR. Table. (DOCX 20 kb) [file 12879_2016_1968_MOESM1_ESM.docx]

**Supplementary table**: Characteristics at cART initiation according to presence/absence of PDR

|  |  | PDR |  | No | PDR |  |
| --- | --- | --- | --- | --- | --- | --- |
| Characteristics |  | n | % | n | % | *P* |
| Sex | Female | 19 | 51.4 | 227 | 51.7 | 1.000 |
| Age (years) | <2 | 14 | 37.8 | 101 | 23.0 | 0.271 |
|  | 2-5 | 9 | 24.3 | 104 | 23.7 |  |
|  | 6-12 | 11 | 29.7 | 186 | 42.4 |  |
|  | 13-17 | 3 | 8.1 | 48 | 10.9 |  |
| Origin | Africa | 8 | 21.6 | 105 | 23.9 | 0.930 |
|  | other/unknown | 5 | 13.5 | 46 | 10.5 |  |
|  | Europe | 9 | 24.3 | 109 | 24.8 |  |
|  | Asia | 15 | 40.5 | 179 | 40.8 |  |
| Transmission risk group | IDU |  |  | 1 | 0.2 | 0.560 |
|  | heterosexual |  |  | 12 | 2.7 |  |
|  | perinatal | 32 | 86.5 | 387 | 8.9 |  |
|  | other/unknown | 5 | 13.5 | 39 | 2.7 |  |
| Previous AIDS diagnosis | YES | 10 | 27.0 | 89 | 20.3 | 0.488 |
|  | No | 27 | 73.0 | 348 | 79.3 |  |
|  | unknown |  |  | 2 | 0.5 |  |
| Pretreatment CD4 count cells/mm^3^ | <50 | 3 | 9.7 | 74 | 19.4 | 0.446 |
|  | ≥50 and <200 | 6 | 19.4 | 73 | 19.2 |  |
|  | ≥200 and <350 | 4 | 12.9 | 73 | 19.2 |  |
|  | ≥350 and <500 | 4 | 12.9 | 37 | 9.7 |  |
|  | ≥500 | 14 | 45.2 | 124 | 32.5 |  |
| Pretreatment VL log_10_copies/mL | <4 | 4 | 11.8 | 32 | 7.7 | 0.624 |
|  | ≥4 and <4.5 | 1 | 2.9 | 46 | 11.1 |  |
|  | ≥4.5 and <5 | 7 | 20.6 | 83 | 20.0 |  |
|  | ≥5 and <5.5 | 10 | 29.4 | 112 | 27.0 |  |
|  | ≥5.5 and <6 | 6 | 17.6 | 86 | 20.7 |  |
|  | >6 | 6 | 17.6 | 56 | 13.5 |  |
| Subtype | NonB | 28 | 75.7 | 354 | 80.6 | 0.454 |
|  | B | 5 | 13.5 | 58 | 13.2 |  |
|  | unknown | 4 | 10.8 | 27 | 6.2 |  |
| Year of treatment start | 1998-1999 | 6 | 16.2 | 99 | 22.6 | 0.120 |
|  | 2000-2002 | 10 | 27.0 | 47 | 10.7 |  |
|  | 2003-2004 | 8 | 21.6 | 104 | 23.7 |  |
|  | 2005-2006 | 10 | 27.0 | 139 | 31.7 |  |
|  | 2007-2008 | 3 | 8.1 | 50 | 11.4 |  |
| Antiretroviral drug combination | NNRTI plus ≥ 2NRTIs^1^ | 254 | 57.0 | 19 | 63.3 | 0.955 |
|  | Unboosted PI plus ≥ 2NRTIs^2^ | 131 | 29.4 | 8 | 37.0 |  |
|  | Boosted PI plus ≥ 2NRTIs^3^ | 41 | 9.2 | 2 | 6.7 |  |
|  | Other^4^ | 20 | 4.4 | 1 | 3.0 |  |
| Number of drugs prescribed | 3 | 31 | 83.8 | 375 | 85.4 | 0.748 |
|  | 4 | 6 | 16.2 | 59 | 13.4 |  |
|  | 5 |  |  | 4 | 0.9 |  |
|  | 6 |  |  | 1 | 0.2 |  |
