# Supplementary material for: Prevalence and effect of pre-treatment drug resistance on the virological response to antiretroviral treatment initiated in HIV-infected children – a EuroCoord-CHAIN-EPPICC joint project
Source: BMC Infect Dis. 2016 Nov 8;16:654. doi: 10.1186/s12879-016-1968-2 (PMC5101717; doi:10.1186/s12879-016-1968-2)
Supplement: Additional file 2: — Crude Hazard Ratios (cHR) and adjusted Hazard Ratios (aHR) for the association between PDR and resistant group versus no PDR/susceptible group from Cox proportional Hazards models stratified by initial cART regimen. HR in the overall group are also presented in more details in Table 3 of the article. All models were adjusted for sex, age, pre-treatment viral load (log10 transformed) and CD4 count, subtype, region of origin, year of treatment start, previous AIDS diagnosis and HIV transmission risk group. (DOCX 46 kb) [file 12879_2016_1968_MOESM2_ESM.docx]

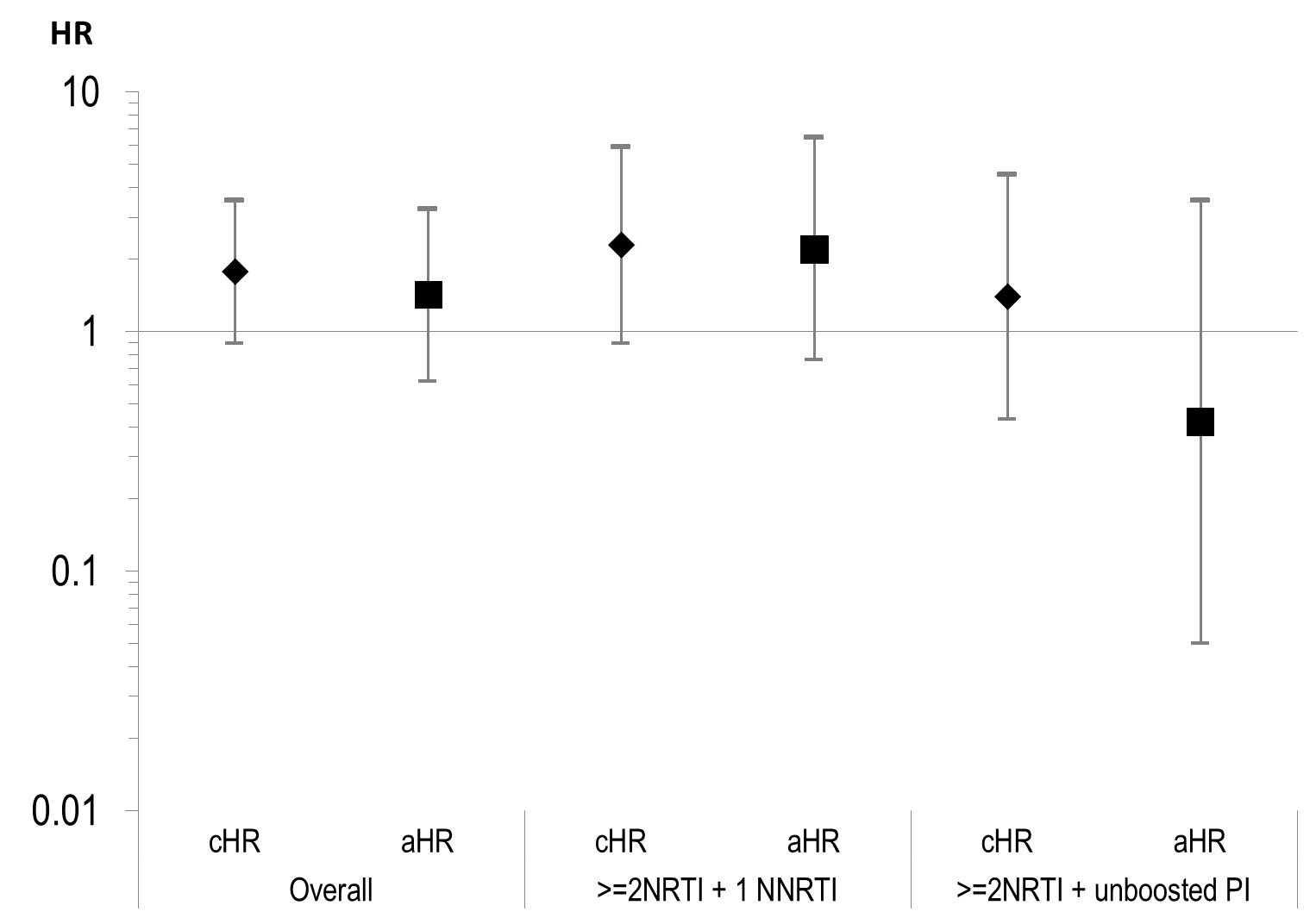


Figure S1: Crude Hazard Ratios (cHR) and adjusted Hazard Ratios (aHR) for the association between PDR and resistant group versus no PDR/susceptible group from Cox proportional Hazards models stratified by initial cART regimen.

HR in the overall group are also presented in more details in table 3 of the article. All models were adjusted for sex, age, pre-treatment viral load (log_10_ transformed) and CD4 count, subtype, region of origin, year of treatment start, previous AIDS diagnosis and HIV transmission risk group.
